# Mechanism‐Aware Digital Twin for High‐Temperature Creep Prediction in Mo–Re Alloys

**DOI:** 10.1002/advs.202509725

**Published:** 2025-09-30

**Authors:** Jinhan Xu, Xuan Chen, Xiaodan Bai, Chengyu Ding, Guisen Liu, Yuanjun Sun, Hongxiang Zong, Xiangdong Ding

**Affiliations:** ^1^ State Key Laboratory for Mechanical Behavior of Materials Xi'an Jiaotong University Xi'an Shaanxi 710049 China; ^2^ State Key Lab of Metal Matrix Composites School of Materials Science and Engineering Shanghai Jiao Tong University Shanghai 200240 China

**Keywords:** creep deformation, digital twin, extreme environments, machine learning, molybdenum–rhenium alloys

## Abstract

Predicting the long‐term deformation of structural materials under extreme conditions remains a grand challenge in materials science, especially for refractory alloys, where high‐temperature creep limits performance and service life. Here, a physics‐informed digital twin framework is developed that integrates a viscoplastic self‐consistent (VPSC) model, real‐time high‐temperature creep experiments, and a calibration neural network to predict and elucidate the creep behavior of Mo‐14Re alloys. The digital twin accurately reproduces creep curves across 1000–1200 °C and 60–150 MPa, achieving <5% deviation from experiments. Crucially, the learned parameter trajectories uncover a previously unrecognized mechanism: Re solute atoms are dragged by gliding dislocations (“solute‐drag” effect), leading to rhenium segregation at grain boundaries and compromised creep strength. This is corroborated by post‐mortem TEM and molecular dynamics simulations. Furthermore, the model‐guided strategy reveals that nanoscale La_2_O_3_ precipitates can pin dislocations and suppress Re segregation, significantly improving creep resistance. This work advances the mechanistic understanding of refractory alloy creep and demonstrates a transferable AI‐enabled digital twin approach for materials design under extreme environments.

## Graphical abstract

A digital twin framework enables real‐time monitoring of creep in refractory alloys and reveals dislocation‐drag‐induced Re segregation to grain boundaries. Inspired by this, La_2_O_3_ dispersion is introduced to suppress dislocation motion — concurrently mitigating Re segregation and enhancing creep resistance.

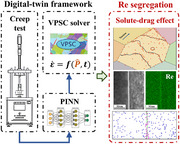

## 1 Introduction

Designing structural materials capable of maintaining mechanical integrity under extreme service conditions—such as high temperatures, sustained loads, and corrosive or irradiative environments—remains a central challenge in materials science.^[^ ^1^, ^2^, ^3^, ^4^ ^]^ These demands are especially critical in aerospace, nuclear, and advanced energy systems, where materials must endure prolonged deformation while preserving microstructural stability.^[^ ^5^, ^6^ ^]^ Despite progress in alloy development, real‐time, mechanism‐aware predictive frameworks are still lacking, limiting our ability to optimize material performance under service‐relevant conditions.^[^ ^6^, ^7^ ^]^ Refractory alloys, in particular, offer high‐temperature strength and thermal stability, yet many—such as body‐centered cubic (BCC) structured metals—suffer from poor room‐temperature ductility and complex creep behavior at elevated temperatures.^[^ ^8^, ^9^, ^10^ ^]^ Mo–Re alloys exemplify these trade‐offs: the addition of rhenium enhances ductility and creep resistance, making them attractive for space nuclear components.^[^ ^11^, ^12^ ^]^ However, their long‐term deformation behavior remains insufficiently understood due to the absence of in situ, time‐resolved experimental and modeling tools that can capture the evolving mechanisms during service.

Historically, high‐temperature creep studies have relied on phenomenological constitutive laws and post‐mortem microscopy. Empirical relations such as Norton's power law^[^ ^13^, ^14^ ^]^ and the *θ*‐projection equation^[^ ^15^, ^16^ ^]^ can reproduce macroscopic strain–time curves but offer limited insight into the evolution and interaction of microscopic deformation mechanisms—such as dislocation glide, solute drag, grain‐boundary sliding, and dynamic recrystallization—during service.^[^ ^16^ ^]^ Some early models (e.g., Nabarro–Herring^[^ ^17^ ^]^ and Coble creep^[^ ^18^ ^]^) incorporated microstructural observations from TEM to interpret creep behavior, providing a physical basis for those empirical parameters. More recently, advances in multiscale modeling and high‐resolution characterization have enabled mesoscale frameworks, particularly crystal plasticity approaches like the viscoplastic self‐consistent (VPSC) model^[^ ^19^, ^20^ ^]^ with Voce hardening,^[^ ^21^ ^]^ to bridge macroscopic creep response and underlying microstructure. However, these models still rely on fixed input parameters—such as dislocation densities, diffusion coefficients, and vacancy concentrations—often derived from averaged pre‐ or post‐test measurements.^[^ ^22^, ^23^ ^]^ As a result, they fail to account for the dynamic evolution of material state during operation, limiting their predictive fidelity.

Digital‐twin (DT) technology—streaming live sensor data into physics‐based models—has recently shown great promise for closing the simulation‐to‐reality gap. Originating in logistics and manufacturing, DTs now improve performance in diverse mechanical and electrical systems.^[^ ^24^, ^25^, ^26^ ^]^ For instance, coupling real‐time feedback from a robotic arm to its kinematic model enhanced path‐tracking accuracy by 15% and cut unplanned downtime by 36%.^[^ ^27^ ^]^ In lithium‐ion batteries, merging operational data with an equivalent‐circuit model reduced the mean‐absolute error of state‐of‐charge predictions to 0.0057 and held synchronization error below 0.2%.^[^ ^28^ ^]^ Building on these successes, DT concepts are beginning to migrate from machinery to multiscale materials modeling—for example, linking process parameters to printed microstructures in additive manufacturing to optimize mechanical properties.^[^ ^29^, ^30^, ^31^ ^]^ These advances suggest that integrating high‐fidelity simulations with in situ microstructural or mechanical measurements could likewise transform the prediction of materials behavior. Yet, unlike mechanical systems with directly measurable control signals, materials science lacks real‐time access to microstructural evolution during processing—posing a critical barrier to the broader adoption of digital twin frameworks in this domain.

Motivated by these considerations, we propose a digital‐twin‐enabled creep modeling framework for Mo–Re alloys, which integrates in situ high‐temperature creep experiments with a VPSC‐based crystal plasticity solver and a physics‐informed calibration neural network. The neural network dynamically adjusts constitutive parameters based on real‐time data while preserving physical consistency. We benchmark the DT framework against conventional fixed‐parameter models and demonstrate its accuracy across a wide range of temperatures and stresses. Furthermore, we exploit the crystal plasticity model's intrinsic interpretability to uncover underlying creep mechanisms—specifically rhenium solute drag and segregation—and evaluate an oxide‐dispersion strengthening strategy using nanoscale La_2_O_3_ precipitates. Beyond the Mo–Re system, the modularity and physics‐awareness of the proposed framework make it readily adaptable to other extreme‐condition materials, such as oxide‐dispersion‐strengthened alloys, high‐entropy alloys, and fusion reactor structural materials. This work thus establishes a generalizable pathway for real‐time, mechanism‐transparent optimization of advanced structural materials under extreme environments.

## 2 Results

### 2.1 Framework of Digital‐Twin‐Enabled Creep Model

**Figure 1:**
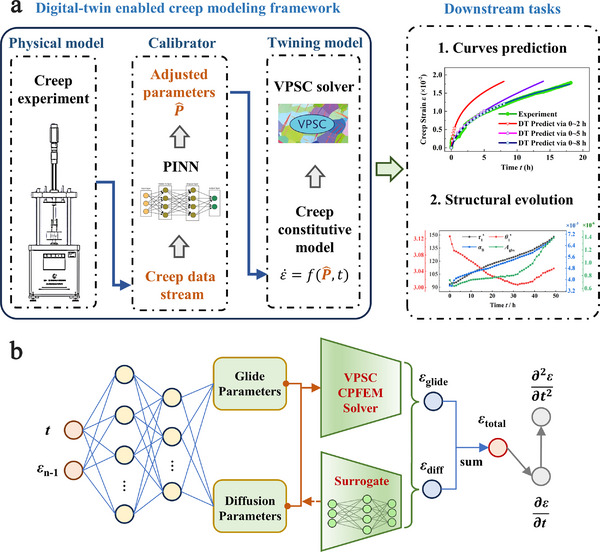
Schematic of digital‐twin enabled creep modeling framework. a) The digital‐twin framework integrates three core components: a viscoplastic self‐consistent (VPSC) crystal‐plasticity solver, in situ high‐temperature creep experiments, and a real‐time parameter calibration. The downstream tasks of digital‐twin framework include prediction of time‐dependent creep strain or strain rate, analysis of creep microstructure evolution and deformation mechanisms, etc. b) Network architecture of our PINN model, which embeds the VPSC solver and its surrogate sub‐NN into the global neural net.

The schematic in **Figure** **1** illustrates the digital‐twin‐enabled creep modeling framework and outlines how it functions to improve the prediction of creep curves. As shown in Figure 1a, our framework integrates three core components: 1) a VPSC crystal‐plasticity solver, 2) in situ high‐temperature creep experiments, and 3) a physics‐informed neural network (PINN) for real‐time parameter calibration. The VPSC solver predicts the evolution of total creep strain, as well as the individual contributions from dislocation glide and diffusional creep, under prescribed loading conditions using a creep constitutive model (see Note S1, Supporting Information). Simultaneously, experimental measurements of creep strain and microstructural state are streamed into the digital twin. The PINN ingests discrepancies between VPSC predictions and experimental data, and adjusts critical constitutive parameters ***P***—including Voce hardening coefficients and diffusion activation energies (see Table S2, Supporting Information)—on‐the‐fly to minimize prediction error. This closed‐loop coupling ensures that the digital twin remains faithful to the actual material behavior throughout the creep test (details see Note S2, Supporting Information). The calibrated model not only yields high‐accuracy creep curves across temperatures and stress levels but also provides interpretable parameter evolution histories that reveal underlying deformation and damage mechanisms.

Within this framework, the PINN, shown in Figure 1b, serves as the key component for dynamically calibrating the VPSC constitutive parameters during the creep simulation. Rather than treating the constitutive parameters ***P*** as fixed, we model them as time‐dependent functions ***P*** (t) to account for microstructural evolution during creep. This choice reflects that, under a given temperature and stress loading, the state of microstructural features (e.g., dislocation density, precipitate distribution, grain‐boundary character) is tightly linked to the dominant creep mechanisms. Any shifts in the prevailing mechanism must be captured through the updating of ***P*** (t). Thus, a fully connected neural network predicts ***P*** (t), which is then passed to the VPSC solver (for forward prediction) or its surrogate (for back‐propagation) to compute total creep strain and its components—dislocation glide and diffusion contributions (Figure 1b). To evaluate the performance of the PINN‐based calibrator, we employ multiple creep curves of Mo‐Re‐based alloys obtained under various loading conditions. The PINN accurately reproduces all experimental creep curves (see Figure S3, Supporting Information), achieving a mean absolute error (MAE) of only 1.52 × 10^−4^, which demonstrates the robustness and predictive capability of the neural network in capturing time‐dependent creep behavior.

**Figure 2:**
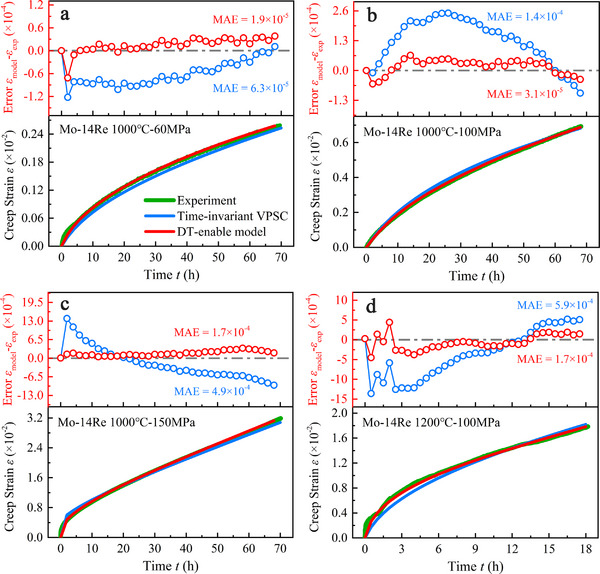
Time evolution of the predicted creep strain curve and error for both time‐invariant VPSC model and our digital‐twin‐enabled creep models. Mo‐14 wt.%Re alloy tested at tensile stress of a) 1000 °C and 60 MPa; b) 1000 °C and 100 MPa; c) 1000 °C and 150 MPa and d) 1200 °C and 100 MPa. The error refers to the difference of predicted stains of models and experimental values.

With a reliable PINN calibrator in hand, we integrated it into a VPSC solver to create the full DT‐enabled creep model. **Figure** **2** compares the DT predictions with those from a conventional, time‐invariant VPSC model and with real‐time experimental measurements. The inset of Figure 2 shows the absolute creep strain at several snapshots; to emphasize the divergence, Figure 2a–d plots the time evolution of the prediction error for both models with different test conditions. Across all conditions, the DT model attains an MAE below 1.7 × 10^−4^, substantially lower than that of the classical VPSC approach. A representative case—Mo‐14 wt.%Re tested at 1200 °C and 100 MPa—illustrates the improvement: the conventional model underestimates strain in the primary stage and overestimates it in the late secondary stage (t >16 h), whereas the DT model, with parameters that evolve continuously in time, tracks the experimental curve throughout. These results underscore the advantage of allowing constitutive parameters to adapt to ongoing microstructural changes, yielding markedly superior predictions of high‐temperature creep deformation.

### 2.2 Interpreting Creep Deformation Mechanisms of Mo‐14Re Alloys

After validating predictive accuracy, we used the digital twin's built‐in parameter tracking to interrogate the deformation mechanisms operating during high‐temperature creep. We focus on Mo‐14Re crept at 1000 °C and 100 MPa—a condition representative of primary and secondary creep. In the underlying VPSC framework, total creep strain is partitioned into two components: i) a dislocation‐glide term governed by Voce hardening, and ii) a diffusion‐controlled term that accounts for vacancy diffusion in both the lattice and at grain boundaries. Because the present VPSC implementation describes only the first two creep stages, the mechanistic discussion that follows is confined to primary and secondary creep.

**Figure 3:**
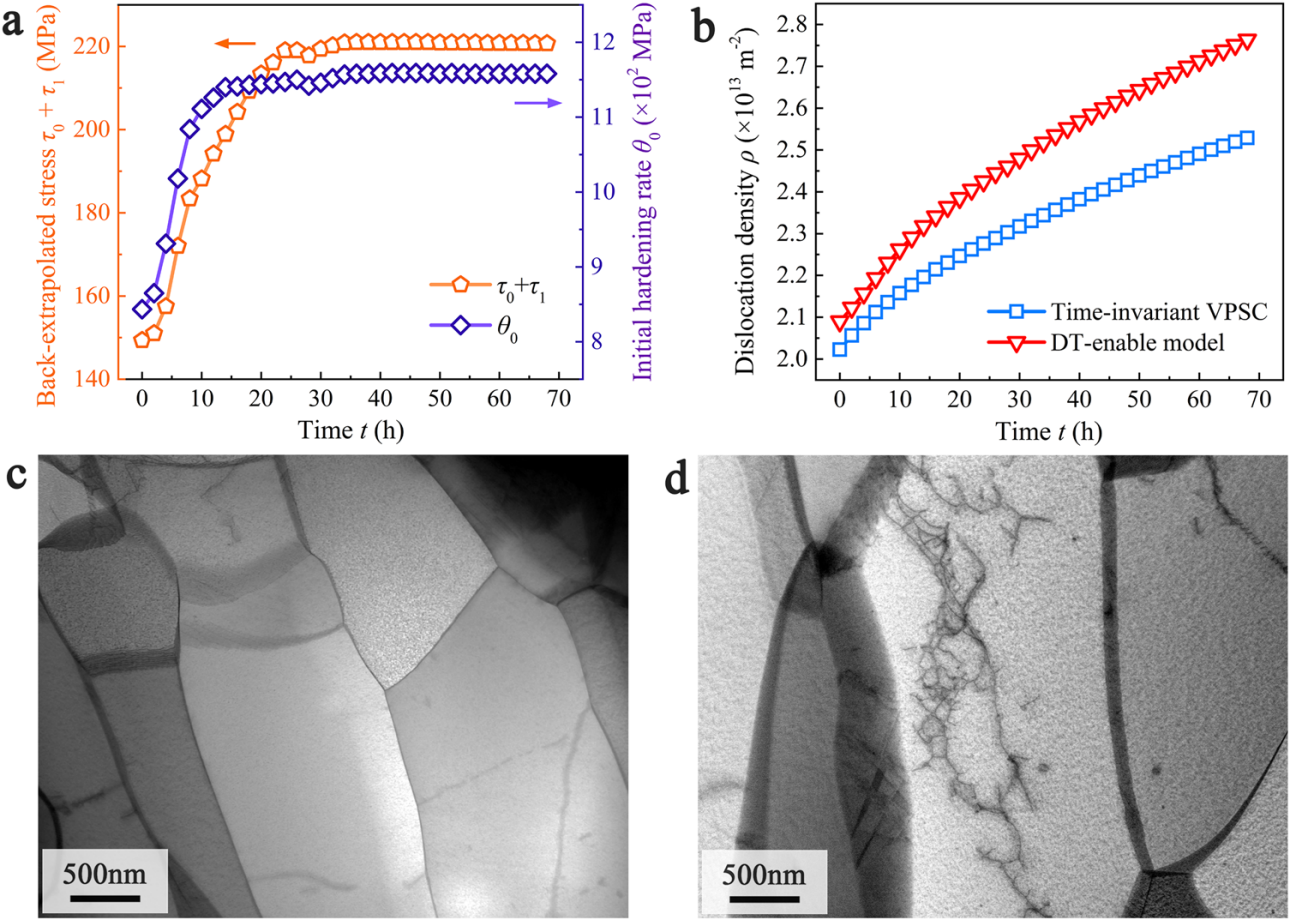
Analysis of dislocation glide mediated term for the creep deformation of Mo‐14 wt.%Re alloys at 100 MPa and 1000 °C. a) The back‐extrapolated stress (*τ*_0_+*τ*_1_) and the initial hardening rate $\theta_{0}^{s}$ extracted from the digital‐twin model. b) Time dependent dislocation density extracted from the digital‐twin model and time‐invariant VPSC. c) and d) Typical TEM pictures obtained from the Mo‐14 wt.%Re alloy test sample before and after 77 h creep deformation.

**Figure** **3**a traces two Voce parameters extracted from the DT model: the back‐extrapolated stress (*τ*_0_+*τ*_1_), which represents the saturation resistance owing to accumulated strain, and the initial hardening rate $\theta_{0}^{s}$. Both (*τ*_0_+*τ*_1_) and $\theta_{0}^{s}$ show a similar trend of initial increase followed by gradual saturation over time. Their combined effect increases the effective resistance to glide, and our analysis below attributes this rise to interactions between dislocations and Re‐rich clusters. Using Equation S3 (Supporting Information), we translate these parameter histories into an equivalent dislocation density, which exhibits a convex, monotonically increasing trend (red color in Figure 3b). For comparison, Figure 3b also plots the dislocation density predicted by a conventional, time‐invariant VPSC model (blue color); the DT framework yields markedly higher values because the real‐time calibration captures the growing back‐extrapolated stress shown in Figure 3a. The elevated dislocation density predicted by the DT model is corroborated by TEM: post‐creep foils contain a far richer dislocation network than the as‐prepared material (Figure 3c,d), confirming that glide is a key contributor to creep. Both models agree that dislocation glide dominates primary creep and, although reduced, remains influential in the secondary stage (see Figure S5, Supporting Information).

**Figure 4:**
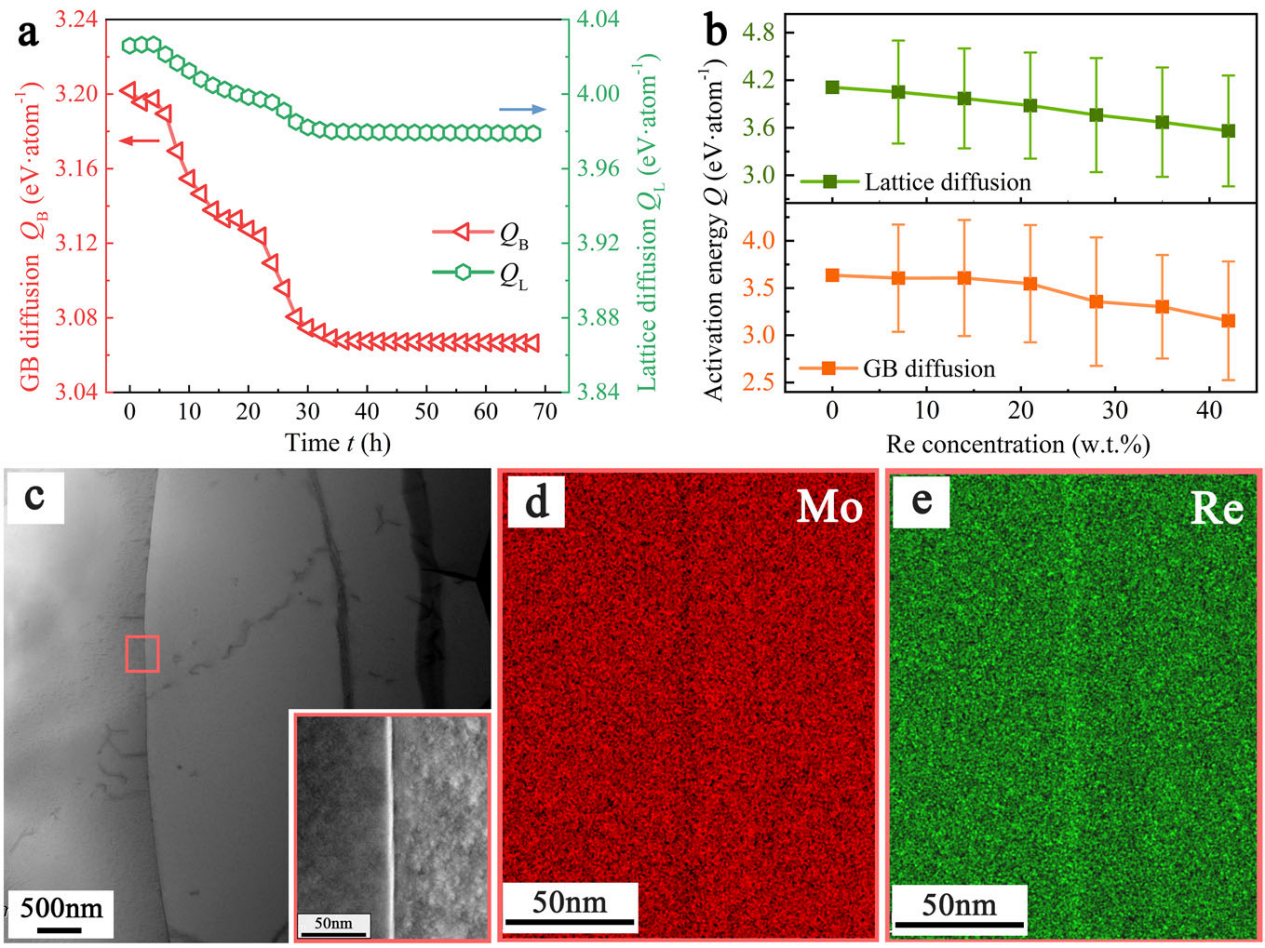
Analysis of diffusion‐mediated term for the creep deformation of Mo‐14 wt.%Re alloys at 100 MPa and 1000 °C. a) The lattice diffusion activation energy *Q*_L_ and GB diffusion activation energy *Q*_B_ traced from the digital‐twin model. b) MD calculation of diffusion energy barrier for Mo‐Re alloy as a function of local Re concentration. c) TEM picture of a typical microstructure after creep and the inset shows a micrograph of a grain‐boundary region enlarged from the red box. d,e) The correspond EDS maps showing the spatial distributions of Mo and Re of the grain‐boundary region.

We next examined the DT parameters that govern diffusion‐controlled creep. **Figure** **4**a tracks the lattice diffusion activation energy *Q*_L_ and GB diffusion activation energy *Q*_B_; both exhibit a rapid decrease within the first 30 h, subsequently approaching asymptotic values of 3.98 and 3.07 eV, respectively. Electron‐backscatter‐diffraction (EBSD) measurements reveal no significant grain‐size change during tensile creep (see Figure S6, Supporting Information), confirming that grain‐boundary migration does not play a primary role under these conditions. This pattern therefore, points to an additional mechanism that accelerates diffusion‐dominated deformation in Mo‐14Re at high temperature. We thus conducted a TEM observation of the creep‐deformed Mo‐14Re samples. The overview micrograph in Figure 4c, and the enlarged region in Figure 4d, reveal a small portion of dislocations terminating at high‐angle GBs after creep at 1000 °C and 100 MPa. Shown in Figure 4e–f, Energy‐dispersive X‐ray spectroscopy (EDS) shows pronounced Re enrichment, with a Re concentration increase of ≈1.8 at. % (Figure S7, Supporting Information), at those boundaries—an observation that is thermodynamically unexpected at such temperatures, pointing to a kinetic driving force. We believe such Re segregation effect is responsible for the time‐dependent diffusion parameters of DT shown in Figure 4a. To test this hypothesis, the activation energy of vacancy‐induced self‐diffusion was calculated with the machine‐learning interatomic potential.^[^ ^32^ ^]^ Figure 4b plots the activation energy for both lattice and GB diffusion as a function of local Re concentration. The MD‐derived activation energies are in good agreement with those tracked from DT model, and the activation energy decreases monotonically with increasing Re content, confirming that Re segregation accelerates atomic transport. These calculations substantiate the time‐dependent reduction of *Q*_L_ and *Q*_B_ in the DT model and rationalize the Re enrichment seen in TEM/EDS maps.

**Figure 5:**
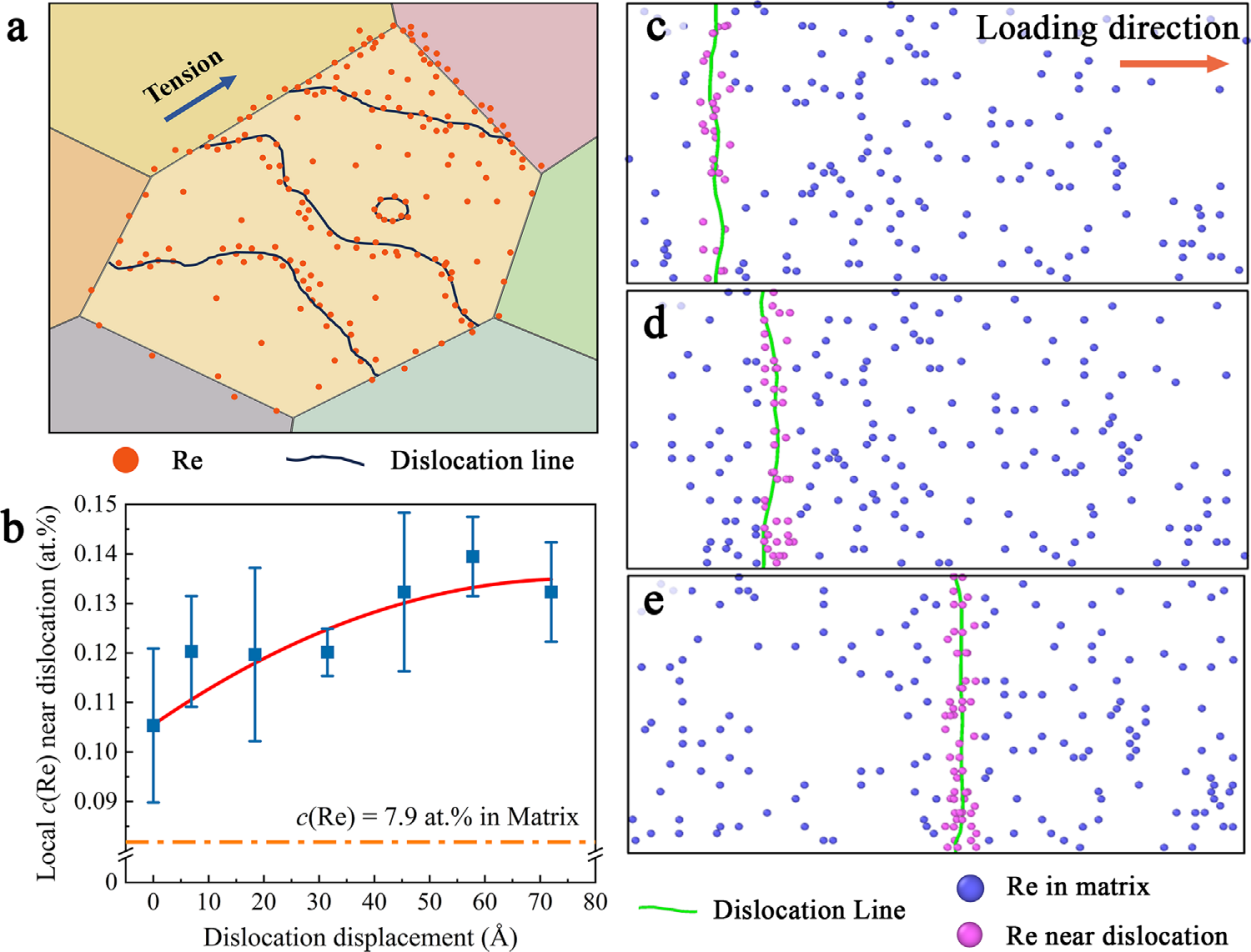
The “Solute‐drag” mechanism for Re segregation at GBs in Mo‐14 wt.%Re alloys. a) Illustration of the “Solute‐drag” mechanism, i.e., dislocation dragged Re atoms migrating toward grain boundaries, b) Re concentration near a moving dislocation line under a constant shear stress loading from MD simulations. c,d,e) The corresponding microstructure evolution of the “Solute‐drag” process.

The atomistic calculations further corroborate that the grain‐boundary Re segregation arises from a dislocation‐mediated solute‐drag mechanism (**Figure** **5**). As schematically illustrated in Figure 5a, at 1000 °C, Re atoms are sufficiently mobile to form atmospheres around moving dislocations owing to their large size misfit with the Mo matrix and the attractive hydrostatic stress of the dislocation core. Order‐of‐magnitude estimates (see Note S10, Supporting Information) place the glide velocity of screw dislocations at ≈1.21 × 10^−10^ m s^−1^, whereas the diffusional velocity of Re atoms into the core region is ≈1.40 × 10^−10^ m s^−1^—the same order of magnitude—leaving ample time for solutes to cluster around the line and move concurrently as it advances. Under applied shear, the dislocation therefore drags its Re atmosphere toward the grain boundary.

MD simulations of a pre‐inserted dislocation in Mo–14 wt.% Re, subjected to a constant resolved shear stress at 1000 °C, confirm this picture. Figure 5b plots the instantaneous Re mole fraction within a cylindrical region of radius 1.5 nm centered on the dislocation line: the Re content rises steadily as the dislocation traverses the Mo‐14Re supercell. Snapshots in Figure 5c–e trace the concurrent microstructural evolution, showing Re‐rich clouds migrating synchronously with the dislocation and depositing at the terminating high‐angle GBs. Together with the characterization and simulation results, these data validate the “solute‐drag” hypothesis and provide a kinetic basis for the time‐dependent diffusion coefficients extracted by the digital twin. Notably, atomic‐scale experimental evidence from Ni‐based single‐crystal superalloys^[^ ^33^ ^]^ also reveals coupling between dislocation cores and Re solute enrichment during creep—offering indirect but compelling support for the solute‐drag mechanism proposed here. Moreover, the clustering of Re atoms—functioning as solute atmospheres—hinders dislocation glide, thereby perturbing the equilibrium between dislocation generation and annihilation. As a result, the overall dislocation density increases, a trend consistent with the observations in Figure 3b. This further corroborates the proposed solute‐drag mechanism.

Notably, the proposed solute drag mechanism becomes significant only when dislocation gliding dominates creep deformation, a scenario commonly associated with low‐temperature and high‐stress creep regimes. Under these conditions, solute atom migration occurs not via intrinsic diffusion, but by coupling with mobile dislocations. Although Re segregation at GBs has also been observed in Mo–Re alloys with similar low Re contents under neutron irradiation conditions,^[^ ^34^, ^35^ ^]^ the mechanism is attributed to the diffusion of self‐interstitial atoms caused by irradiation‐ a process that is largely absent in conventional deformation or thermal conditions. In a similar vein, A similar solute redistribution behavior has been reported in 304 stainless steel, attributed to the migration of solute atoms toward grain boundaries driven by vacancy flux.^[^ ^36^ ^]^ Such effects were observed under high‐temperature (> 0.6 *T*_m_), low‐stress (< 20 MPa) creep conditions, where vacancy diffusion governs the deformation process. In contrast, the present study focuses on a distinct segregation mechanism that operates under dislocation‐mediated creep conditions, highlighting the role of stress and deformation mode in determining solute segregation pathways.

### 2.3 Suggesting Strategies for Mitigating The “Solute‐Drag” Effect

Our DT analysis reveals that the intensity of the solute‐drag effect scales with dislocation mobility; hence, immobilizing dislocations with fine precipitates should kinetically suppress Re segregation at grain boundaries. Rare‐earth oxides—especially La_2_O_3_—are known both to raise the high‐temperature strength of Mo alloys and to preserve acceptable room‐temperature ductility.^[^ ^37^ ^]^ Motivated by this, we prepared an oxide‐dispersion‐strengthened (ODS) Mo‐14Re alloy containing nano‐scale La_2_O_3_ and evaluated its creep response with the DT framework.

**Figure 6:**
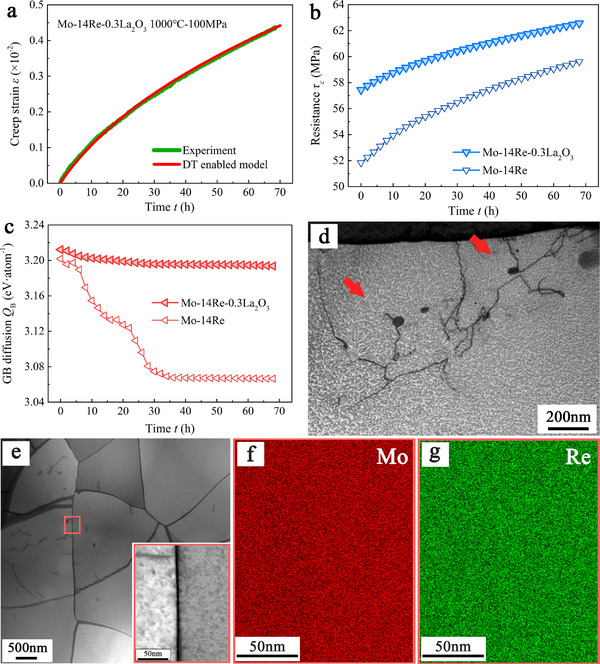
Analysis of Creep Microstructure Evolution in La_2_O_3_‐Doped Mo–14Re Alloys at 100 MPa and 1000 °C. a) Digital twin (DT) prediction of the time‐dependent creep strain curve, showing good agreement with experimental data. b,c) Corresponding evolution of b) dislocation gliding resistance and c) grain boundary (GB) diffusion activation energy (*Q*_B_) tracked from the digital twin model. d) TEM bright‐field image showing dislocation pinning by La_2_O_3_ particles. Red arrows indicate representative pinning events. e) TEM micrograph of a representative microstructure after creep deformation. The inset shows an enlarged view of a grain boundary region, corresponding to the red box indicated in the main image. f,g) EDS elemental maps illustrating the spatial distributions of Mo f) and Re g) across the selected grain boundary region.

**Figure** **6** summarizes the results for tensile creep of Mo‐14Re‐0.3La_2_O_3_ alloys at 1000 °C and 100 MPa. The DT prediction faithfully reproduces the experimental strain–time curve (Figure 6a). Then, the microstructural evolution between doped and undoped Mo–14Re alloys is comparatively analyzed to elucidate the mechanistic role of La_2_O_3_ dispersion. The resistance to dislocation gliding (Figure 6b), as reflected by the tracked Voce parameters, is significantly higher than that in the undoped alloy, indicating that the La_2_O_3_ provides additional strengthening via the Orowan mechanism. This interpretation is further supported by TEM imaging (see Figure 6d), which directly reveals dislocations pinned at the La_2_O_3_ particles. This increased resistance further implies a reduced dislocation velocity, which is consistent with the lower creep strain observed in the experiment (Figure 6a). In contrast, although La_2_O_3_ dispersion generates a high density of dislocations during the creep deformation (see Figure S10a, Supporting Information), the activation energies for lattice and GB diffusion, as tracked by the DT model, exhibit only a minor reduction upon La_2_O_3_ doping (Figure 6c; Figure S10b, Supporting Information) compared to the substantial decrease observed in the absence of La_2_O_3_. This demonstrates that the solute‐drag process is effectively curtailed by the presence of La_2_O_3_. Collectively, these findings demonstrate that La_2_O_3_ dispersion mitigates Re segregation by restricting dislocation motion and therefore provides a viable route to improving the creep resistance of Mo–Re alloys.

TEM observations of the La_2_O_3_‐doped Mo‐14Re alloy further corroborate the absence of Re segregation at the original (high‐angle) grain boundaries and clarify the associated recovery processes. After 66 h of creep testing at 1000 °C and 100 MPa, the microstructure exhibits a dense dislocation network within the grain interiors. Meanwhile, EDS mapping across high‐angle grain boundaries (Figure 6e–g) reveals no detectable rhenium (Re) enrichment – a finding consistent with the statistically significant reduction in the average Re concentration increase, which drops from 2.0 ± 0.27 at.% in the Mo‐14Re alloy to merely 0.43 ± 0.31 at.% in the La_2_O_3_‐doped alloy (see Figure S7, Supporting Information). The accumulation of dislocations is expected because La_2_O_3_ nanoparticles pin glide and promote dislocation pile‐ups; the resulting stored energy provides a strong driving force for dynamic recovery and particle‐stimulated recrystallization. Indeed, TEM frequently captures dislocation rearrangement into low‐angle sub‐boundaries and the emergence of fine, particle‐encircled subgrains—features characteristic of recovery and recrystallization induced locally around oxide particles rather than along the pre‐existing high‐angle boundaries considered in our model (see Figure S11, Supporting Information). Dynamic recovery mainly proceeds through dislocation–dislocation reactions and does not transport solute to the original grain boundaries, while the newly formed low‐angle boundaries generated near La_2_O_3_ particles are too short and discontinuous to serve as long‐range solute sinks. Consequently, the absence of Re segregation at high‐angle boundaries cannot be attributed to lattice diffusion; vacancy‐mediated bulk diffusion and pipe diffusion along pinned dislocations would, in that case, still result in detectable Re enrichment. The EDS evidence therefore supports our primary conclusion: dispersed La_2_O_3_ particles suppress the dislocation‐mediated solute‐drag pathway and, in turn, inhibit Re segregation at the grain boundaries most critical to creep rupture.

## 3 Conclusion

In this study, we present a real‐time materials digital twin framework that integrates meso‐scale modeling with dynamic AI calibration to predict high‐temperature creep behavior in Mo–Re alloys. By coupling a viscoplastic self‐consistent model with real‐time experimental data and a physics‐informed neural network for on‐the‐fly parameter adjustment, this framework shifts from static, phenomenological predictions to a dynamic, mechanism‐aware approach. This advancement enables more accurate and adaptable creep predictions, capturing the evolution of material states during testing and offering deeper insights into material behavior under extreme conditions.

Our approach not only improves understanding of Mo–Re alloy creep, particularly through the solute‐drag effect leading to rhenium segregation at grain boundaries, but also reveals that La_2_O_3_ dispersion effectively suppresses high‐temperature creep. This finding suggests that rare‐earth oxide doping may serve as a transferable strategy to enhance creep resistance in other lightweight refractory alloys. Furthermore, the digital twin framework has been demonstrated as a powerful platform for materials optimization in harsh environments. By further incorporating microstructural phenomena such as grain growth, phase stability, and irradiation damage, the framework's predictive capabilities can be further enhanced for more comprehensive lifetime performance assessments under complex service conditions. Looking ahead, integrating this platform with autonomous experimentation and real‐time sensing offers the opportunity to close the loop between modeling, testing, evaluating, and alloy design, accelerating the discovery of next‐generation structural materials that can endure the most demanding conditions in aerospace and energy applications.

## 4 Experimental Section

### Statistical Analysis

The data in this manuscript can be divided into three main sections: i) time‐series creep data and ii) molecular dynamics (MD) simulation results. As for the time‐series data, the mean absolute error (MAE) between the model‐predicted curve and the experimental values is defined as:

(1) $$\begin{array}{c} MAE=\frac{1}{n}\;\sum_{n}\left|x_{\mathrm{model}}-x_{\exp}\right| \end{array}$$

where *n* is the number of discrete data points and *x* represents the physical quantity being measured (including creep strain and strain rate). Mean relative error (MRE) is used to describe the relative deviation, and its definition is given as:

(2) $$\begin{array}{c} MRE=\frac{1}{n}\;\sum_{n}\left|\frac{x_{\mathrm{model}}-x_{\exp}}{x_{\exp}}\right| \end{array}$$

During MD simulation, the diffusion activation energy at grain boundaries and within the lattice, as well as the solute concentration near dislocations, were each calculated ten times to estimate their mean value and sample standard deviation.

## Conflict of Interest

The authors declare no conflict of interest.

## Author Contributions

J.X. and X.C. contributed equally to this work. J.X. curated data, performed formal analysis and visualization, developed methodology, worked with the software, and wrote the original draft. X.C. curated data curation, performed investigation, and wrote the original draft. X.B. curated data and developed the methodology. C.D. performed formal analysis and worked with the software. G.L. developed the methodology and worked with the software. Y.S. acquired resources. H.Z. conceptualized the study, acquired funds and resources, performed supervision, and wrote, reviewed, and edited the final draft. X.D. conceptualized the study and performed projection administration and supervision. All the authors discussed the results and commented on the manuscript.

## Supporting information



Supporting Information

## Data Availability

The data that support the findings of this study are available from the corresponding author upon reasonable request.
