# Supplementary material for: Mechanism‐Aware Digital Twin for High‐Temperature Creep Prediction in Mo–Re Alloys
Source: Adv Sci (Weinh). 2025 Sep 30;12(48):e09725. doi: 10.1002/advs.202509725 (PMC12752588; doi:10.1002/advs.202509725)
Supplement: Supplementary file 1 — Supporting Information [file ADVS-12-e09725-s001.pdf]

Supporting Information to Mechanism-Aware Digital Twin for High-Temperature Creep Prediction in Mo–Re Alloys

Jinhan Xu†, Xuan Chen†, Xiaodan Bai, Chengyu Ding, Guisen Liu, Yuanjun Sun, Hongxiang Zong, Xiangdong Ding**

†These authors have contributed equally to this work.

Supplementary Note 1 | Crystal plasticity-based creep model solved by VPSC approach.

In the present work, we develop a crystal plasticity-based creep model to describe the creep deformation behavior of Mo-Re alloys. It is solved by the VPSC approach proposed by Tome et. al.[1,2] In this model, the creep rate includes the contribution of dislocation glide and diffusion, that is

$$\begin{cases} \dot{\varepsilon}_{\mathrm{glide}} = \sum_{\mathrm{s}} m_{\mathrm{ij}}^{\mathrm{s}}\, \dot{\gamma}_0 \left(\frac{\tau^{\mathrm{s}}}{\tau_{\mathrm{c}}^{\mathrm{s}}}\right)^n \\ \dot{\varepsilon}_{\mathrm{diff}} = \left(\frac{\alpha_{\mathrm{B}}}{d^3} + \frac{\alpha_{\mathrm{L}}}{d^2}\right)\sigma \end{cases} \tag{S1}$$

where the gliding creep rate $\dot{\varepsilon}_{\mathrm{glide}}$ is computed as the tensor sum of the slip rates on all slip systems. $\dot{\gamma}_0$ represent reference slip rate and n stands for the inverse of the rate sensitivity exponent. For each slip systems, $m_{\mathrm{ij}}^{\mathrm{s}}$ is Schmid factor, τ^{s} is resolved shear stress (RSS) that is calculated by the applied load and Schmid factor, and $\tau_{\mathrm{c}}^{\mathrm{s}}$ is the resistance of dislocation gliding. Here, the resistance $\tau_{\mathrm{c}}^{\mathrm{s}}$ was described by Voce hardening[3], i.e.,

$$\tau_{\mathrm{c}}^{\mathrm{s}}(\Gamma) = \tau_0^{\mathrm{s}} + (\tau_1^{\mathrm{s}} + \theta_1^{\mathrm{s}}\Gamma)\left(1 - \exp\left(-\Gamma\frac{\theta_0^{\mathrm{s}}}{\tau_1^{\mathrm{s}}}\right)\right) \tag{S2}$$

In Equation S2, Γ is the accumulated strain of all slip systems and $(\tau_0^{\mathrm{s}}, \tau_1^{\mathrm{s}}, \theta_0^{\mathrm{s}}, \theta_1^{\mathrm{s}})$ are the four Voce parameters. In this sense, the corresponding dislocation density could be estimated from Voce hardening parameters (see Supplementary Note 15) by

$$\sqrt{\rho} = \frac{\tau_0^{\mathrm{s}}}{\alpha G b} + \frac{\tau_1^{\mathrm{s}} + \theta_1^{\mathrm{s}}\Gamma}{\alpha G b}\left[1 - \exp\left(-\frac{\theta_0^{\mathrm{s}}}{\tau_1^{\mathrm{s}}}\Gamma\right)\right] \tag{S3}$$

where α is the hardening factor of dislocation forests, G is shear modulus and b is Burgers vector.[4] If the alloy contains second-phase particles, i.e. La_2O_3 ODS, dislocations is impeded by the particles with an extra resistance, given by dispersed-barrier hardening model[5], shown in Equation S4:

$$\tau_{\mathrm{O}} = \alpha_{\mathrm{O}} G b \sqrt{N D_{\mathrm{P}}} \tag{S4}$$

where α_O is the hardening factor of second-phase particles, N is number density of the particles and D_p represents their average diameters.

The diffusion creep rate $\dot{\varepsilon}_{\text{diff}}$ in Equation S1 is related to the contribution of vacancy diffusion, and it can be divided by two parts, i.e., the lattice diffusion creep (coble model) and boundary diffusion creep (Nabarro-Herring model).[6,7] The corresponding diffusion parameters α_B and α_L can be achieved by

$$\begin{cases} \alpha_L = \dfrac{A_L D_L^0 \Omega}{3kT} \exp\left(-\dfrac{Q_L}{kT}\right) \\ \alpha_B = \dfrac{A_B D_B^0 c\Omega}{kT} \exp\left(-\dfrac{Q_B}{kT}\right) \end{cases} \quad (S5)$$

where A_L and A_B are constant factors of lattice and boundary creep. D_L^0 and D_B^0 are preexponential of diffusion coefficients of vacancy in lattice or near the grain boundary. Q_L and Q_B are the correspond activation energies of the two diffusion coefficients. Ω is atomic volume and c is the thickness of the boundary.

Table S1 lists the constant parameters of the creep constitutive model, while **Table S2** provides the variable parameters calibrated by DT framework. For the dislocation gliding term, Voce parameters were chosen as adjustable values to track the evolution of dislocation density. Correspondingly, for the diffusion term, the activation energies for lattice and grain boundary diffusion were selected as the two adjustable parameters to quantify the diffusion intensity, as well as solute distribution in the Mo-Re alloy.

Table S1 The constant parameters the creep constitutive models.

No.	Fixed parameter	Equation	Value
1	$\dot{\gamma}_0$ (Reference slip rate)	(S1)	6.1×10^{-3} h^{-1}
2	*n* (Stress sensitive factor)	(S1)	7
3	*d* (Average grain size)	(S1)	20 μm
4	*α* (hardening factor of dislocation forests)	(S3)	0.5
5	*G* (Shear modulus)	(S3)	(118 – 0.0272*T*) GPa [8]
6	*b* (Burgers vector)	(S3)	2.72×10^{-9} m [8]
7	α_O (hardening factor of La_2O_3 particles)	(S4)	0.5
8	*N* (number density of La_2O_3)	(S4)	2.83×10^{17} m^{-3}
9	D_p (average diameter of La_2O_3)	(S4)	150 nm
10	A_L (Lattice creep factor)	(S5)	15 [9]
11	D_L^0(Preexponential of lattice diffusion)	(S5)	5.0×10^{-4} $m^2\cdot s^{-1}$
12	*Ω* (Atomic volume)	(S5)	1.97×10^{-29} m^3
13	*k* (Boltzmann constant)	(S5)	1.38×10^{-23} m^3
14	A_B (Boundary creep factor)	(S5)	158 [9]
15	D_B^0 (Preexponential of boundary diffusion)	(S5)	1.0×10^{-3} $m^2\cdot s^{-1}$
16	*c* (Thickness of the boundary)	(S5)	2.0×10^{-9} m

Table S2 Creep constitutive parameters calibrated by DT framework.

No.	Time-dependent parameter	Mo-14Re	Mo-14Re-0.3La_2O_3
1	τ_0^s (Initial CRSS for all slip systems)	51.8 MPa	55.4 MPa
2	$\tau_0^s + \tau_1^s$ (The back-extrapolated stress)	140 MPa	170 MPa
3	θ_0^s (Initial hardening rate)	840 MPa	1190 MPa
4	θ_1^s (Initial hardening rate)	2 MPa	2 MPa
5	Q_B (GB diffusion activation energy)	3.20 $eV\cdot atom^{-1}$	3.21 $eV\cdot atom^{-1}$
6	Q_L (Lattice diffusion activation energy)	4.03 $eV\cdot atom^{-1}$	4.03 $eV\cdot atom^{-1}$

Supplementary Note 2 | Technical specifics of digital twin system workflow.

We have incorporated a workflow diagram illustrating the closed-loop calibration process. Within this digital twin framework (**Figure S1**), the creep experiment transmits a data batch to the system every ΔT_c (8 hours in our study). The digital twin system then polls for incoming data at regular intervals of ΔT (1 hour). Upon detecting new data, the PINN calibrator is activated to update the constitutive model parameters (see Supplementary Note 3). In the absence of new data, the system employs the current parameter set to forecast the subsequent creep curve segment and simulate microstructural evolution. During the loop, VPSC steps (~2.6 s), PINN epochs (~112.4 s), and full PINN updates (~20 min) all complete well within the one-hour polling interval, ensuring negligible computational latency and eliminating any workflow disruption.

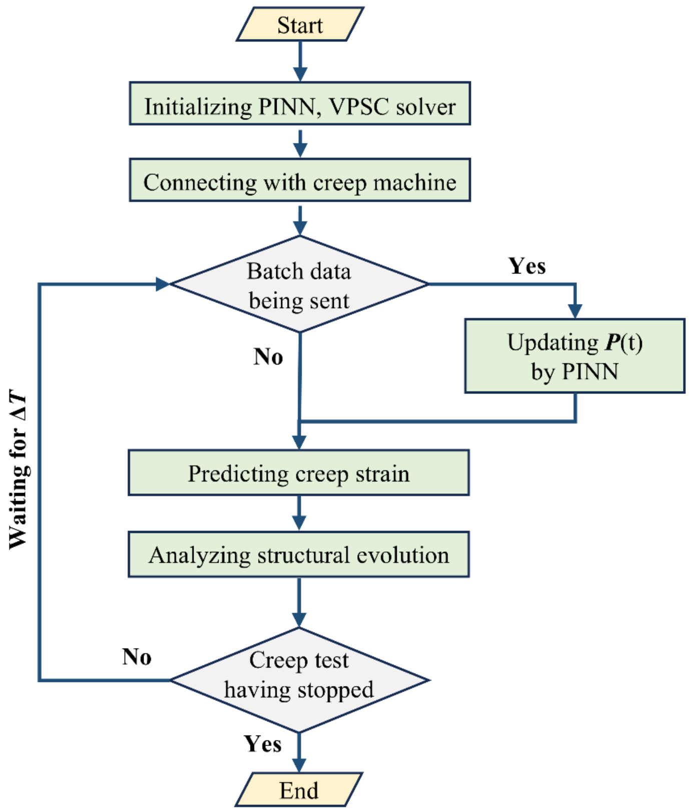

Figure S1. Schematic of the digital twin workflow.

Supplementary Note 3 | Details of real-time parameter calibration.

We employ a physics-informed neural network (PINN)[10] to dynamically calibrate the VPSC constitutive parameters. The PINN contains a fully connected neural network to predicts ***P***(t), then embedded VPSC solver (for forward prediction) together with its surrogate (for back-propagation) to compute creep strain. The neural network is trained on in situ creep data via the loss, i.e.,

$$L = \alpha \mathrm{MSE}(\hat{\varepsilon}_{\mathrm{net}}, \varepsilon_{\mathrm{exp}}) + \mathrm{MSE}(\hat{\dot{\varepsilon}}_{\mathrm{net}}, \dot{\varepsilon}_{\mathrm{exp}}) + \sum \mathrm{ReLU}(-\hat{\ddot{\varepsilon}}_{\mathrm{net}}) \quad \text{(S6)}$$

where the last two terms were added to avoid overfitting by controlling the monotonicity and non-convexity of the creep strain curve. Here, $\hat{\varepsilon}_{\mathrm{net}}$ and $\varepsilon_{\mathrm{exp}}$ denote strains from automatic differentiation and experiments, respectively; $\hat{\dot{\varepsilon}}_{\mathrm{net}}$ and $\hat{\ddot{\varepsilon}}_{\mathrm{net}}$ are first and second order gradients of predicted strain via back-propagation; α represent an adjustable parameter, $\mathrm{MSE}(x, y) = \frac{1}{N}\sum(x-y)^2$ and $\mathrm{ReLU}(x) = \max(x, 0)$.

There are two fully connected neural networks in the PINN calibrator (Figure 1b) in the main text), the network mapping from time to constitutive parameters consists of fully connected layers with 1, 54, 72, 36, 12, and 6 neurons, respectively; the NN surrogate model comprises layers with 6, 72, 48, 16, and 3 neurons. All layers use LeakyReLU activation functions with negative slope being 0.01. The training hyperparameters are: learning rate of 0.0001, 30 epochs, and a batch size of 16.

The complete details of the digital-twin-enabled creep model and the code for the PINN implementation have been uploaded to https://github.com/XujinhanMSE/Digital-twin-enabled-Creep-Framework/tree/master.

Supplementary Note 4 | Experimental and MD simulation details.

4.1 Experimental details

The Mo-14Re alloys as well as La_2O_3 particles doped Mo-14Re alloys were prepared by powder metallurgy. The mixed powders of Mo-0.3 wt % La_2O_3 powder (purity ≥ 99.95 %, mean size ≈ 4.8 μm) and high-purity Re powder (purity ≥ 99.99 %, mean size ≈ 40 μm) were obtained by the S-S doping method to obtain a Mo-14 wt % Re mixture. This is followed by a mechanical milling process, i.e., the powders were ball-milled for 48 h in a sealed stainless-steel jar (polyurethane liner) at 50 rpm under argon, using a ball-to-powder mass ratio of 1:1. The milled powders were then cold-isostatically pressed into 50 mm-diameter cylinders, vacuum-sintered at 1900 °C for 6 h, and hot-forged at 1300 °C to 25 mm-diameter rods. A final anneal at 1000 °C for 1 h in flowing H_2 removed residual stresses.

Creep specimens (dimensions: 6 × 3 × 30 mm) were prepared by electrical discharge machining and then surface-ground to minimize the influence of machining defects. Creep tests were performed on an UZDL-2500 machine under specified temperature and tensile-stress conditions, continuing until fracture or interruption after 100 h. During each test, a video extensometer recorded the specimen's real-time creep strain and streamed the data directly into the real-time parameter calibration module. The creep testing machine is equipped with built-in signal filtering to ensure inherently smooth creep data. A Savitsky-Golay filter is employed, with a window size of 21 data points and a second-order polynomial fit.

Microstructures before and after creep testing were examined by transmission electron microscopy (TEM, FEI Talos F200X). Localized deformation substructures and dynamic recrystallization were analyzed via transmission Kikuchi diffraction (TKD, Oxford C-Nano+). TEM/TKD foils were prepared by twin-jet electropolishing in 12.5 vol % H_2SO_4–ethanol at −15 °C under 10 V.

4.2 MD simulations

To probe Re–dislocation interactions during high-temperature creep, we performed MD simulations on BCC Mo-14wt.%Re single-crystal solids containing pre-inserted dislocation lines. The interatomic interactions within the Mo-Re binary alloys were described by our previously developed machine-learning potential, which faithfully reproduces Re segregation, point-defect migration, and dislocation behavior in Mo–Re alloys.[11] Simulation cells (dimensions: 18 nm) were first equilibrated at 1000 K for 100 ps under an isothermal–isobaric (NPT) ensemble. Thereafter, a constant shear stress was applied along the [111] direction, concurrently with short-range Monte Carlo steps to accelerate solute redistribution. Additionally, the activation energies Q_L and Q_B for lattice and grain boundary self-diffusion were calculated using a 10 nm cubic supercell, either with or without a Σ5(310) grain boundary. The values were determined by summing the corresponding vacancy migration barriers E_b and formation energies E_f, as derived from Equation S7.[12]

$$Q = E_{\mathrm{b}} + E_{\mathrm{f}} \tag{S7}$$

The vacancy diffusion barriers were computed using zero-temperature nudged-elastic-band (NEB) method. All runs were carried out using LAMMPS.[13]

Supplementary Note 5 | Prediction performance of PINN calibrator.

The training loss evolution of the PINN calibrator is plotted in **Figure S2**. The loss decreases monotonically throughout optimization, with each training run converging robustly to a final value of approximately 0.005 — indicating stable and effective convergence without signs of stagnation or overfitting.

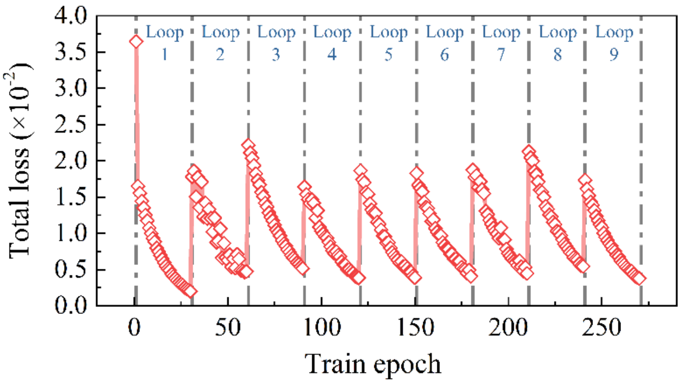

Figure S2. training loss evolution of PINN calibrating of Mo-14wt%Re crept at 1000°C and 100MPa.

Following confirmation of the PINN calibrator's convergence, we first assessed its ability to reproduce the creep response of Mo-Re alloys. **Figure S3** summarizes the results for several Mo-Re-based alloys tested under different loading histories. In Figure S1a, each data point—taken from the experimental creep curves at selected stresses and times—compares the measured creep strain with the PINN prediction for Mo-14 wt.%Re. The agreement is excellent, with a mean absolute error (MAE) of only 1.52×10^{-4}. The strain-rate comparison in Figure S1b is similarly accurate with a smaller MAE of 7.76×10^{-5} h^{-1}. We next examined the PINN in an auto-regressive mode, allowing it to generate full strain and strain-rate histories. As shown in Figures S1c-f, the creep curves under different loading conditions are captured faithfully, demonstrating the neural network's robustness in forecasting time-dependent creep behavior. Notably, the monotonicity and convexity constraint embedded in the loss function (see Equation S5) suppresses spurious features—such as the artificial acceleration that would otherwise appear for t >17 h in Figure S1f — thereby preserving physical realism while maintaining high predictive accuracy.

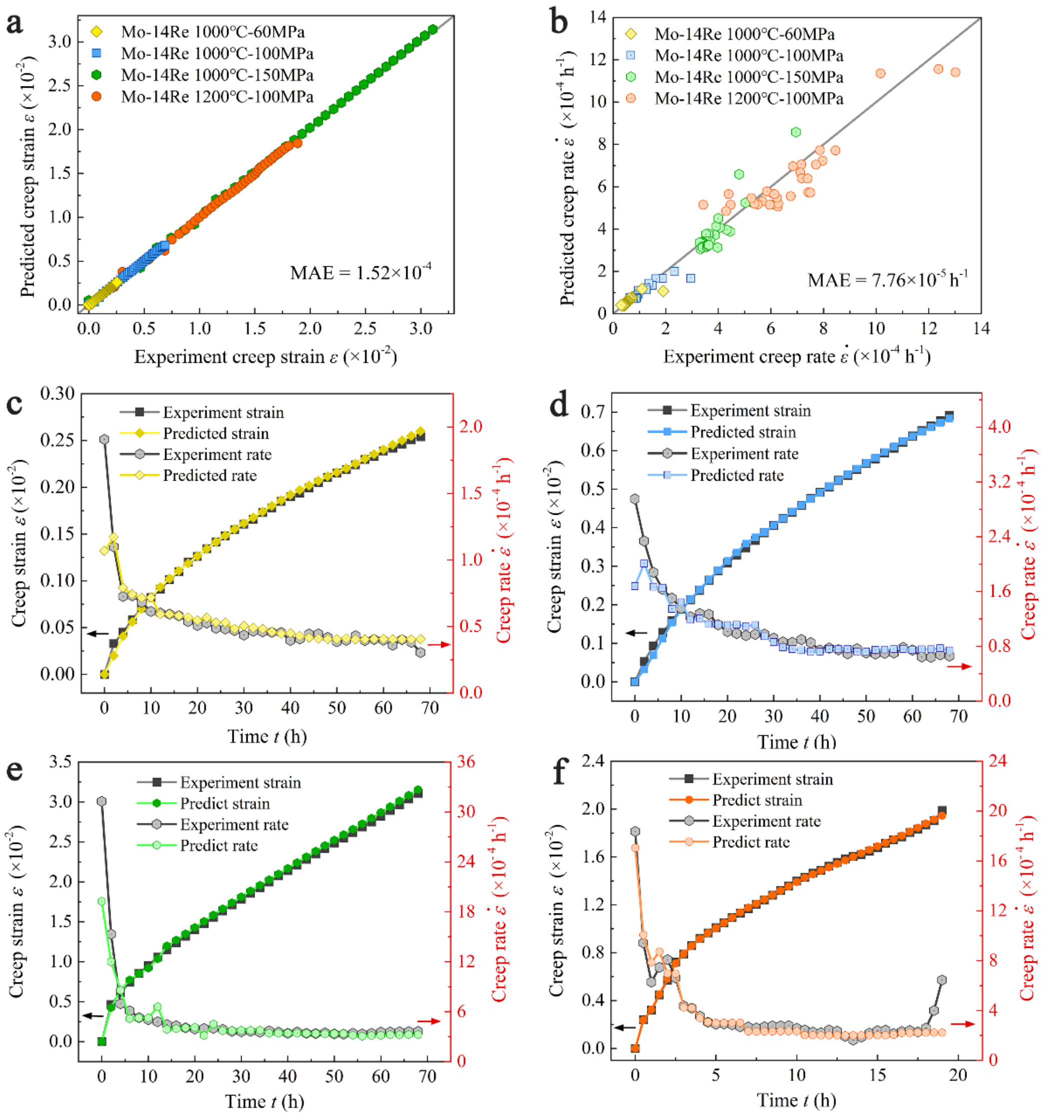

Figure S3. Creep deformation properties predicted by the PINN calibrator model compared with the experimental results. a) discrete strain data, b) discrete strain-rate data, c) 1000 °C and 60 MPa; d) 1000 °C and 100 MPa; e) 1000 °C and 150 MPa and f) 1200 °C and 100 MPa creep curves for Mo-14 wt.%Re alloy.

Supplementary Note 6 | Sensitivity analysis of PINN calibrator.

To evaluate the sensitivity of the PINN to input noise, we introduced Gaussian noise into the experimental creep data of Mo-14wt.%Re alloy at 1000 °C and 100 MPa using the method defined in Equation S8.

$$\hat{\varepsilon} = \varepsilon(1 + \Delta x), \qquad \Delta x \sim \frac{\sigma}{\varepsilon} N(0, 1) \tag{S8}$$

where $\hat{\varepsilon}$ is the creep strain gaussian noise while ε is the original strain without noise. Δx represents the relative error, which follows a normal distribution with standard deviation σ/ε – commonly referred to as the coefficient of variation.

As shown in **Figure S4**, even as noise intensity increases, the MAE of the PINN-predicted curves remains remarkably stable – consistently on the order of 10^{-4} (Figure S4a) – demonstrating strong robustness. Visual comparisons across multiple noise-contaminated datasets (Figures S4b-d) demonstrate that the PINN architecture inherently suppresses high-frequency fluctuations while accurately capturing the intrinsic evolution of the creep response – a direct consequence of its physics-constrained regularization. This resilience stems from the physics-informed constraints embedded in the PINN architecture and the inherent smoothing effect of the VPSC model's regularization. Notably, our experimental setup further enhances data fidelity: the creep testing machine itself incorporates built-in filtering, actively suppressing high-frequency noise and preserving curve continuity.

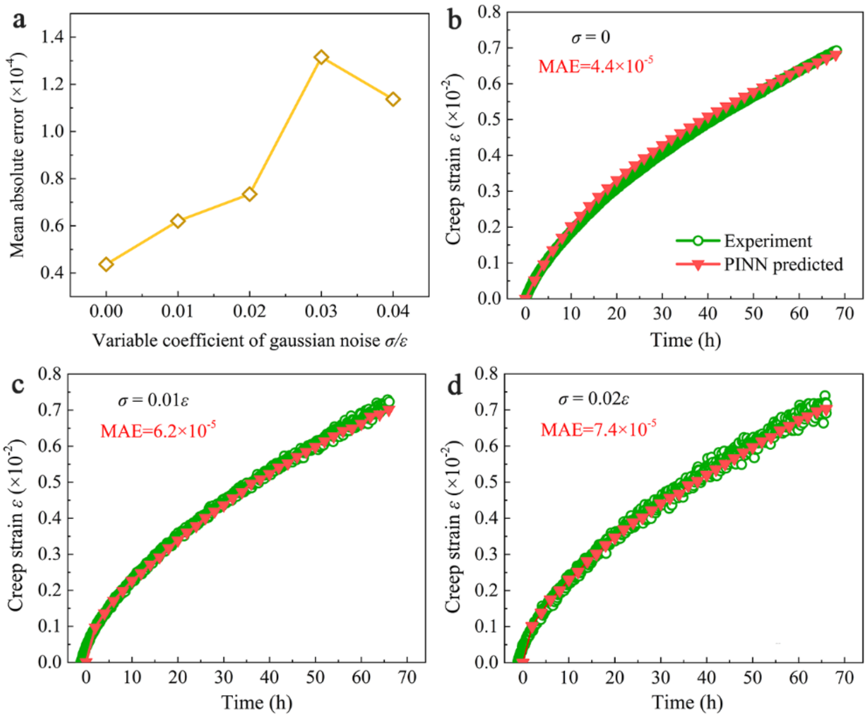

Figure S4. Sensitivity analysis of PINN calibrator. a) model prediction accuracy with intensity of gaussian noise in Mo-14wt%Re alloy crept at 1000°C and 100MPa. Creep properties comparison of PINN predictions gaussian-noised experimental curves at different variance of normal distribution b) $\sigma = 0$, c) $\sigma = 0.01\varepsilon$ and d) $\sigma = 0.02\varepsilon$.

Supplementary Note 7 | Creep mechanism contribution comparison: time-invariant vs. digital twin-enabled models.

The creep curve of the Mo-14wt.%Re alloy under 1000 °C and 100 MPa was selected as a representative instance to compare the contributions of creep mechanisms between the traditional time-invariant model and the proposed DT-enabled model. As shown in **Figure S5**, dislocation gliding dominates the deformation process in both models, contributing more than 90% to the total strain. In contrast, the contribution from diffusion creep increases significantly in the DT-enabled model–approximately 2.5 times higher than that in the time-invariant model–indicating the activation of the diffusion mechanism during creep deformation.

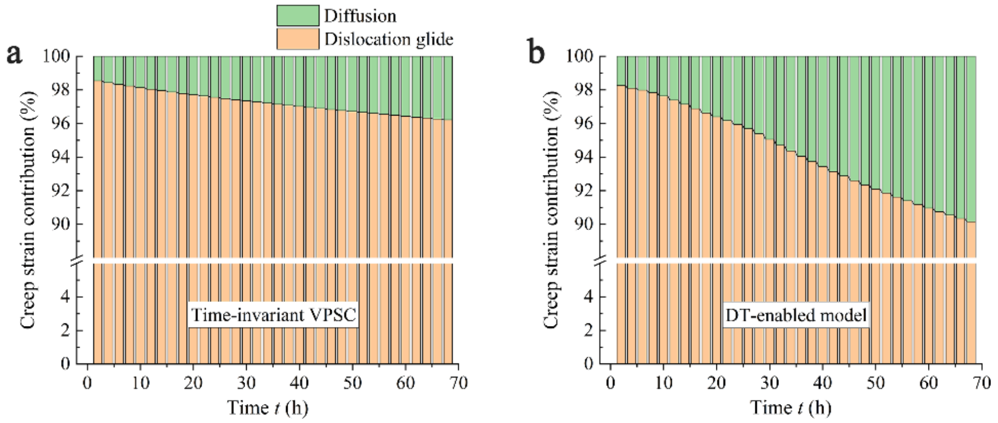

Figure S5. Contributions of dislocation glide and diffusional mechanisms to the total creep strain in the Mo-14wt.% Re alloy tested under a tensile stress of 100 MPa at 1000 °C. a) Time-invariant VPSC model; b) DT-enabled model.

Supplementary Note 8 | Evidence against grain growth as the origin of Re segregation.

The activation of the diffusion creep mechanism in the Mo-14wt.%Re alloy was attributed to Re segregation at grain boundaries (see Section 2.2 in the main text). To investigate whether this segregation was associated with grain growth, EBSD characterization was conducted to measure the average grain size before and after creep deformation. The results (see **Figure S6**) revealed no significant difference in grain size after 66 hours of creep, suggesting that the observed Re segregation is not caused by grain growth but rather by some other underlying mechanism.

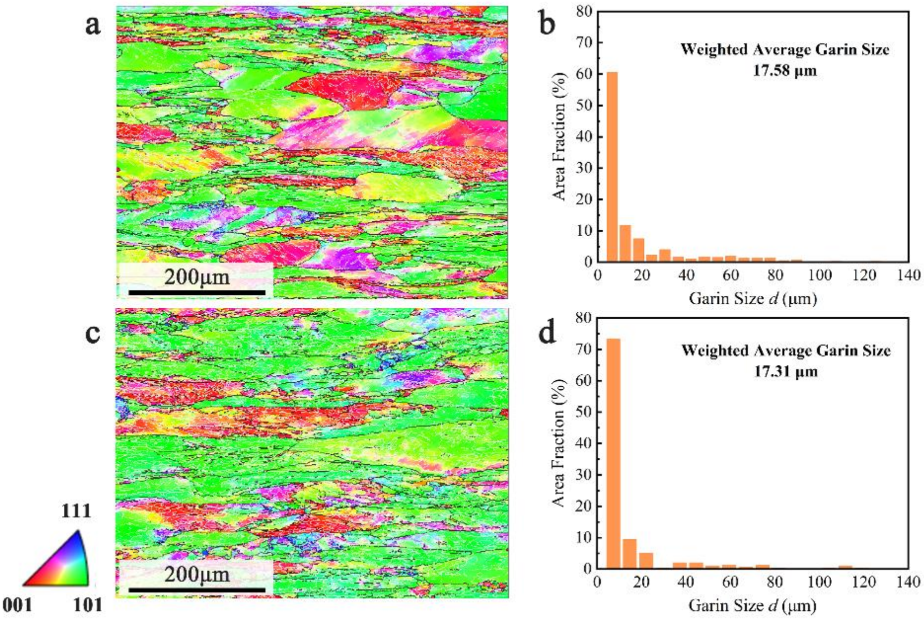

Figure S6. EBSD maps of the Mo-14Re alloy a) prior to and c) following creep deformation at 1000 °C and 100 MPa. High-angle grain boundaries (>10°) are marked by black lines. b) and d) Display the corresponding grain size distributions before and after creep, respectively.

Supplementary Note 9 | EDS line-scan analysis of Re segregation at grain boundaries in Mo-14Re and Mo-14Re-0.3La_2O_3 alloys.

To accurately quantify the Re segregation observed in EDS maps, multiple line scans were performed across grain boundaries (GBs). The results of these scans are presented in **Figure S7**. Specifically, Figures S7a-c illustrate the segregation analysis for Mo-14Re alloys, corresponding to Figures 4c-e in the main text. Re exhibits a pronounced enrichment at the GB relative to the matrix—a clear signature of Re segregation. Based on the line scan results shown in Figure S7c, we introduced a parameter, termed "Re enrichment factor", to quantify the degree of Re segregation at GBs[14], as described by Equation S9:

$$\Delta c(\mathrm{Re}) = c_{\mathrm{GB}}(\mathrm{Re}) - \bar{c}_{\mathrm{Matrix}}(\mathrm{Re}) \qquad (S9)$$

Here, $c_{\mathrm{GB}}(\mathrm{Re})$ represents the Re concentration at the GB, while $\bar{c}_{\mathrm{Matrix}}(\mathrm{Re})$ is the average Re concentration in the matrix, calculated as the arithmetic mean of the signal from positions [0, 40] nm and [60, 100] nm along the line scan. These intervals were chosen to avoid regions directly affected by the GB and ensure an accurate representation of the matrix composition. Using the definition, the Re enrichment at the selected boundary in Mo-14Re alloy is calculated to be 1.8 at.%. Similarly, the Re concentration distributions across grain boundaries (GBs) of the Mo-14Re-0.3La_2O_3 alloy were also analyzed and are presented in Figures S7d-f, corresponding to Figures 6e-g in the main text. The analysis reveals that Re exhibits negligible enrichment at the grain boundaries, with a concentration increase of only 0.21 at.% relative to the matrix. This indicates that there is no significant segregation of Re at these interfaces.

Multiple EDS line scans were performed in both Mo-14Re and Mo-14Re-0.3La_2O_3 alloys, and the Re enrichment factors were statistically analyzed (n = 5). As shown in Figure S7g, the average Re enrichment factor in Mo-14Re is 2.0 ± 0.27 at.%, whereas in Mo-14Re-0.3La_2O_3 it drops significantly to 0.43 ± 0.31 at.%. This marked reduction demonstrates that La_2O_3 effectively suppresses Re segregation at grain boundaries. These quantitative results provide

strong support for the proposed solute drag mechanism, confirming its role in mitigating Re partitioning.

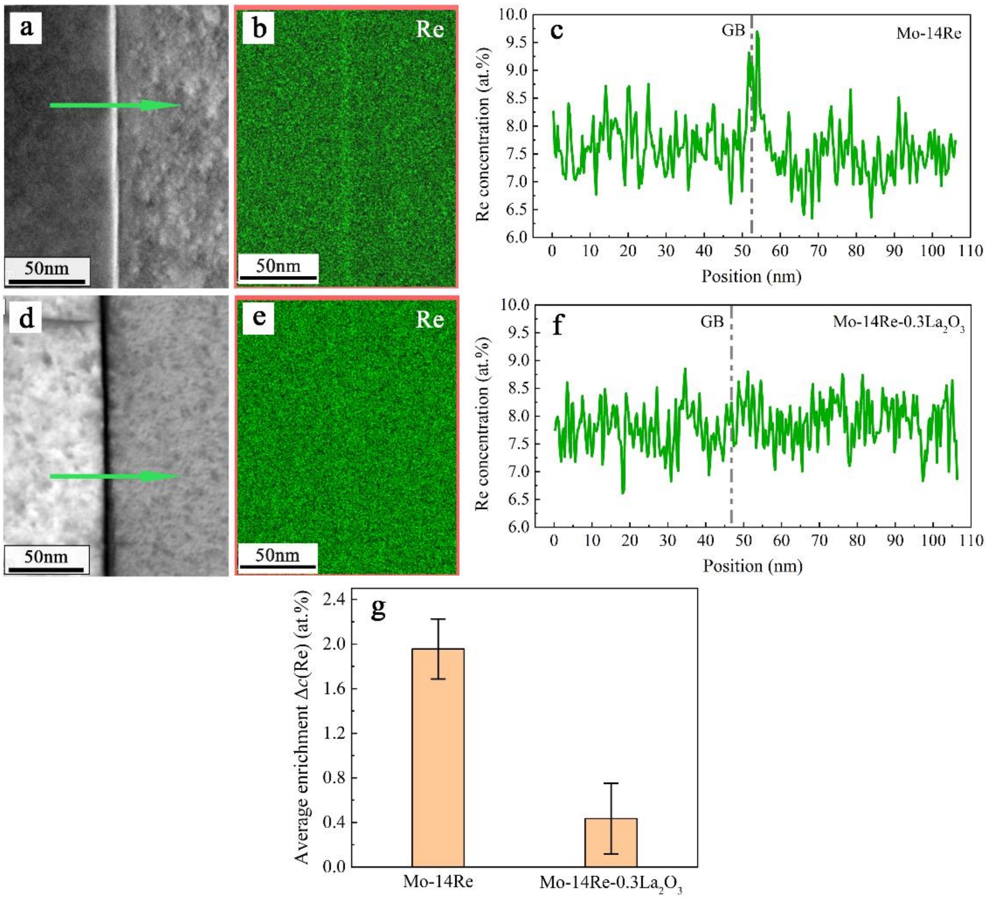

Figure S7. EDS analysis of Re segregation at grain boundaries (GBs) in a-c) Mo-14Re and d-f) Mo-14Re-0.3La_2O_3 alloy after creep deformation. a) and d) the views of a grain boundary region. b) and e) elemental distribution maps of Re across the selected GB. c and f) Re concentration profile obtained via EDS line scan across the GB. g) Statistical comparison of Re enrichment at GBs in Mo–14Re and Mo-14Re-0.3 La_2O_3 alloys, with $\Delta c(\mathrm{Re}) = c_{\mathrm{GB}} - c_{\mathrm{matrix}}$.

Supplementary Note 10 | Order-of-magnitude estimates for dislocation glide velocities and Re atom diffusion rates.

Based on classical diffusion and dislocation theories, we estimate the order of magnitude of both dislocation motion and Re atom diffusion rates induced by dislocation-induced stress fields. In general, the average rate of an atom in the lattice is given by Equation S10 [15,16]:

$$v = \frac{D}{kT} \cdot F \tag{S10}$$

where the *D* represents for the self-diffusion coefficient of the corresponding element, *k* is Boltzmann constant and *T* is thermodynamic temperature. *F* is the average driven force on the solute atom. Since the Re atom is influenced by the dislocation, we utilize the "mismatch sphere" model to describe the interaction force between the solute atoms and the dislocation, as expressed in Equation S11[17].

$$F = \frac{Gb(1+v)\Delta V}{3\pi(1-v)} \cdot \frac{1}{r^2} \tag{S11}$$

where *G* is shear modulus, *b* is Burgers vector, *v* is Poisson's ratio and ΔV is the mismatch volume of the solute atoms. ΔV is estimated by atomic radius of Mo and Re via Equation S12 [17].

$$\Delta V = \frac{4}{3}\pi(r_{\mathrm{Re}}^3 - r_{\mathrm{Mo}}^3) \tag{S12}$$

The values of the physical quantities were computed using MD simulations, that is: G = 89.8 GPa, b = 2.72 Å, v = 0.304, r_{Mo} = 1.35 Å and r_{Re} = 1.40 Å. We consider the attractive effect on Re atoms within a cylindrical region of 10 Å radius centered at the dislocation, and thus set the characteristic distance r = 5 Å. The self-diffusion coefficient is 9.04×10^{-20} $\mathrm{m^2 \cdot s^{-1}}$ through DT-enabled creep model under 1273 K (see Figure 6 in the main text). Using Equation S10-12, the average velocity of Re atoms in the vicinity of the dislocation is estimated as v_{Re} = 1.40×10^{-10} $\mathrm{m \cdot s^{-1}}$.

On the other hand, the average dislocation glide velocity can be derived from the strain rate and dislocation density based on Orowan equation [4,18], as follows:

$$\dot{\varepsilon} = \rho v b \tag{S13}$$

Our creep test of Mo-14Re under 1000° C and 100 MPa, combined with the DT-enabled creep model, has yielded its average strain rates $\dot{\varepsilon}$ = 0.9×10^{-4} h^{-1} and dislocation density ρ = 7.6×10^{11} m^{-2}. It can be concluded that the average dislocation glide velocity is v_{glide} = 1.21×10^{-10} m·s^{-1}.

Based on the above results, it can be inferred that during the high-temperature creep of Mo-14Re, the diffusion rate of Re atoms is on the same order of magnitude as the dislocation glide velocity observed during plastic deformation, and may even be slightly faster. Such a result suggests that Re atoms may migrate alongside dislocations during plastic deformation. Thus, while mobile dislocations continuously migrate and annihilate at grain boundaries, they may simultaneously carry Re atoms to these regions. The consequent alterations in the chemical environment at the grain boundaries can affect the material's diffusion coefficients, leading to an enhancement of Coble creep. As a result, we propose a hypothesis of "solute drag" effect in this study, with supporting evidence provided by both experimental observations and simulation results shown in Figures 4 and 5 of the main text.

Moreover, it should be noted that the cooperative migration between Re atoms and dislocations cannot be detected during uniaxial tensile tests. This is because the average strain rate in such tests is on the order of 10^{-3} s^{-1}, leading to a dislocation glide velocity of approximately 10^{-5} m·s^{-1}. Given that glide velocity is significantly higher than the diffusion rate of Re atoms, it is unlikely that dislocations can effectively transport the solute atoms during their movement. Therefore, such solute drag phenomena are likely to occur only during creep or high-temperature slow-strain-rate tensile tests.

Supplementary Note 11 | Stress-enhanced vacancy diffusion in Mo–14Re alloy revealed by MD simulations.

To verify the phenomenon of stress-enhanced diffusion in Mo-14Re alloy, we calculated the diffusion coefficients under both thermodynamic equilibrium and applied tensile stress conditions using molecular dynamics (MD) simulations. As shown in **Figure S8**, the diffusion coefficient – corresponding to vacancy-mediated diffusion – was determined by computing the mean square displacement (MSD) in a 14×14×14 supercell containing a single pre-introduced vacancy, under varying applied stresses, according to Equation S14[19].

$$D_{\mathrm{b}} = \frac{1}{6}\lim_{t\to\infty}\frac{\langle r^2(t)\rangle}{t} \tag{S14}$$

The results reveal that the vacancy diffusion coefficient increases under applied stress, providing indirect support for the stress-enhanced activation of Re diffusion.

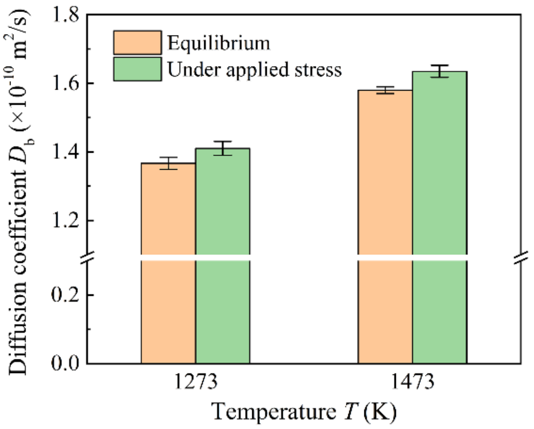

Figure S8. Vacancy diffusion coefficient of Mo-14Re alloys at different applied stress.

Supplementary Note 12 | Microstructural evolution in Mo-14Re-0.3La_2O_3 alloys during creep.

Expect for Figure 6d in the main text, other TEM image also provides clear evidence of La_2O_3 particles effectively pinning dislocations within the Mo-14Re-0.3La_2O_3 alloy, shown in **Figure S9**. The image reveals numerous dislocations entangled around La_2O_3 particles, with red arrow indicating several representative instances of this pinning effect. These observations support the role of La_2O_3 in pinning dislocations as discussed in section 2.3 in the main text.

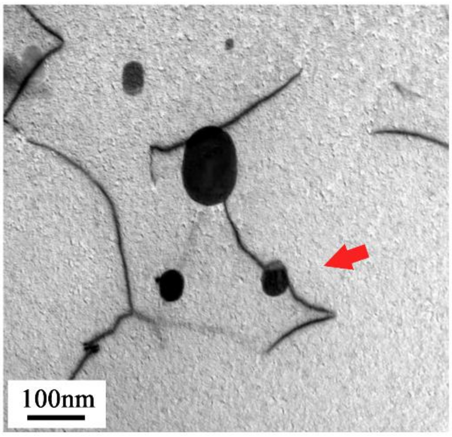

FigureS9. TEM images showing La_2O_3 particles pinning dislocations in the Mo-14Re-0.3La_2O_3 alloy. Red arrows highlight several typical examples of dislocation pinning at different scales.

The evolution of dislocation density in the Mo-14Re-0.3La_2O_3 alloy during creep at 1000 °C and 100 MPa was analyzed based on the tracked Voce parameters. Using Equation S4 from the main text, the resistance to dislocation motion imposed by La_2O_3 particles was calculated to be 2.36 MPa. This contribution was subsequently subtracted from the total resistance (Figure 6b in the main text), and the remaining component was converted into dislocation density, as shown in **Figure S10**a. For comparison, the dislocation density of the La_2O_3-free Mo-14Re alloy is also presented in the same figure. The results indicate that the addition of La_2O_3 leads to a higher dislocation density, suggesting a reduction in dislocation mobility.

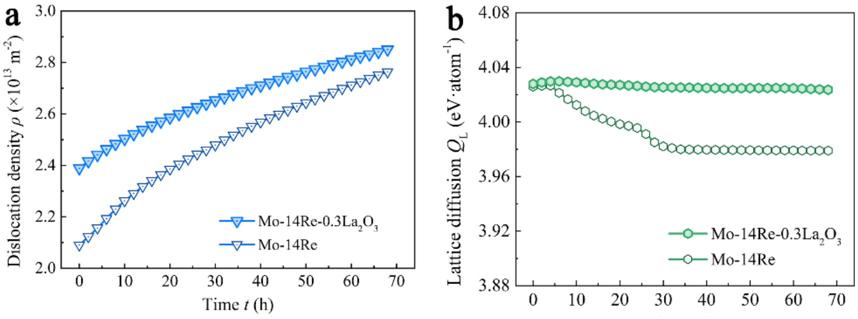

Figure S10. Microstructural evolution of Mo-14Re-0.3La_2O_3 alloy during creep at 1000 °C and 100 MPa tracked by DT models. a) Density dislocation ρ; b) Lattice diffusion activation energy Q_L.

As shown in Figure S10b, the activation energies for lattice diffusion follow the same trend as those for grain boundary (GB) diffusion (Figure 6c): only a minor reduction is observed upon La_2O_3 doping, in contrast to the substantial decrease seen in its absence. This suggests that the addition of La_2O_3 suppresses Re segregation — consistent with the predictions of our proposed solute-drag mechanism.

Supplementary Note 13 | Crystallographic characterization of grain boundary segregation after creep deformation.

The crystallographic orientations of selected grains in the Mo-14Re-0.3La_2O_3 alloy after creep deformation were analyzed using transmission Kikuchi diffraction (TKD) mapping, shown in **Figure S11**. The observed grain boundary—highlighted within the red box and corresponding to the region shown in Figure 6e of the main text—was identified as a high-angle grain boundary (HAGB) (see Figure S11b). This indicates that the boundary was present prior to creep loading and is not a sub-boundary formed during recrystallization. Consequently, the segregation behavior at this HAGB is representative for evaluating the interaction between Re distribution and creep deformation.

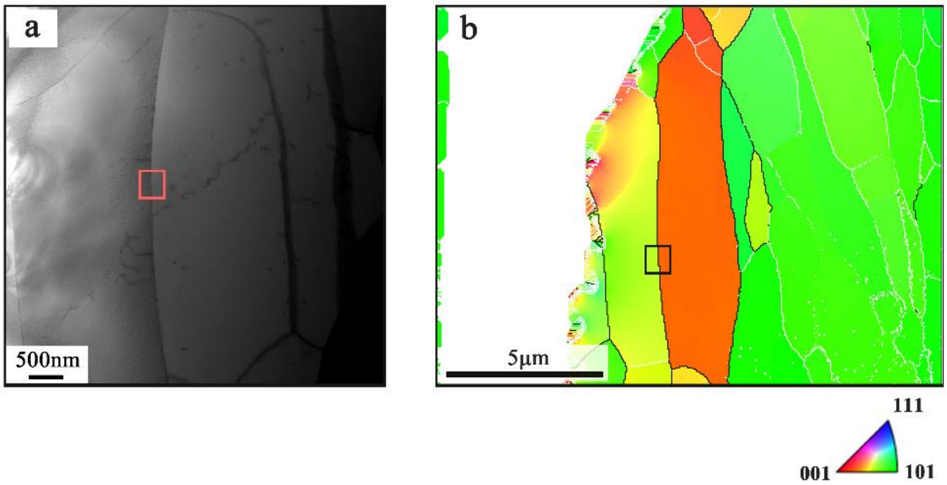

Figure S11. TEM characterization of the typical microstructure of Mo-14Re alloy after creep at 1000°C and 100MPa. a) Bright-field image and b) TKD mappings. The red box in a) and the black box in b) correspond to the same region. High-angle grain boundaries (>10°) are marked by black lines in b).

Supplementary Note 14 | Size stability of La_2O_3 during the creep process.

To assess the thermal stability of La_2O_3 nanoparticles and exclude the risk of coarsening during creep, particle sizes were measured via TEM both before and after creep deformation, shown in **Figure S12**. Statistical analysis of over 40 particles reveals that, after 77 h of creep at 1000 °C, the average particle size increased only slightly — from 60 ± 23 nm to 67 ± 24 nm. This marginal growth (~12%) confirms that La_2O_3 nanoparticles exhibit excellent thermal stability under the investigated creep conditions and do not undergo significant coarsening at 1000 °C.

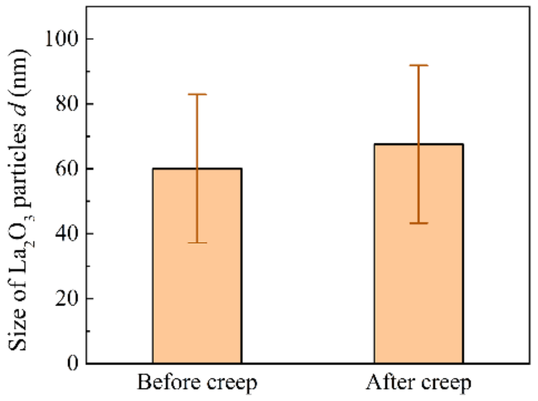

Figure S12. Size distribution of La_2O_3 particles before and after 66 hours creep deformation in Mo-14Re-0.3La_2O_3 alloy.

Supplementary Note 15 | Derivation of dislocation density evolution formula based on Voce hardening parameters.

The Voce hardening law is derivate from dislocation density (DD) evolution during the plastic deformation. The average dislocation distribution could be describe by Kocks‑Mecking formulation (Equation S15) showing the DD rate is the sum of a storage term and an annihilation term[4].

$$\frac{d\rho}{d\varepsilon} = k_1\sqrt{\rho} - k_2\rho \tag{S15}$$

where k_1 and k_2 are the storage and dynamic recovery coefficients, respectively. Then, the Taylor law (Equation S16) shows the yield stress σ is proportional to the squared-root of dislocation density[3].

$$\sigma = \alpha G b\sqrt{\rho} \tag{S16}$$

By combining the Equation S15 and S16, a differential equation of flow stress and strain during plastic deformation can be summarized as Equation S17.

$$\frac{d\sigma}{d\varepsilon} = k_1' - k_2'\sigma \tag{S17}$$

Here, k_1' and k_2' are two coefficient factors, related to k_1 and k_2 as shown in Equation S18.

$$\begin{cases} k_1' = \dfrac{\alpha G b}{2} k_1 \\ k_2' = \dfrac{k_2}{2} \end{cases} \tag{S18}$$

Based on Equation S17, the flow stress as a function of accumulated strain was derived and expressed as Equation S19, which corresponds to the Voce hardening law formulated in terms of overall scalar magnitudes[20].

$$\sigma(\varepsilon) = \sigma_0 + \sigma_1\left(1 - \exp\left(-\frac{\theta_0\varepsilon}{\sigma_1}\right)\right) \tag{S19}$$

where σ_0, (σ_0 + σ_1) and θ_0 are the initial stress, back-extrapolated flow stress and initial hardening rate, respectively. The relationship between those parameters (σ_0, σ_1, θ_0) and (k_1', k_2') is seen in Equation S20.

$$\begin{cases} k_1' = \theta_0 \left(\dfrac{\sigma_0}{\sigma_1} + 1 \right) \\ k_2' = \dfrac{\theta_0}{\sigma_1} \end{cases} \tag{S20}$$

The widely used Voce hardening law for slip system scale, developed by Tome et al.[20,21], is shown as Equation S21.

$$\hat{\tau}^s = \tau_0^s + (\tau_1^s + \theta_1^s \Gamma)\left(1 - \exp\left(-\Gamma \frac{\theta_0^s}{\tau_1^s}\right)\right) \tag{S21}$$

where τ_0^s, $(\tau_0^s + \tau_1^s)$, θ_0^s and θ_1^s are Voce parameters, representing the initial threshold stress, back-extrapolated flow stress, initial hardening rate and the asymptotic hardening rate, respectively. Γ denotes the accumulated shear strain of all slip systems. This equation is essentially consistent with Equation S19, except that the overall parameters have been transformed to represent values across all slip systems. These substitution relationships are detailed in Equation S22.

$$\begin{cases} \sigma_0 \to \tau_0^s \\ \sigma_1 \to \tau_1^s + \theta_1^s \Gamma \\ \theta_0 \to \theta_0^s \end{cases} \tag{S22}$$

In our work, the Voce parameters have been tracked from digital-twin frameworks. To capture dislocation density evolution during the creep deformation, we can connect Voce parameters to the storage and dynamic recovery coefficients k_1 and k_2 through Equation S18, S20 and S22, that is:

$$\begin{cases} k_1 = \dfrac{2\theta_0^s}{\alpha G b}\left(\dfrac{\tau_0^s}{\tau_1^s + \theta_1^s \Gamma} + 1 \right) \\ k_2 = \dfrac{2\theta_0^s}{\tau_1^s + \theta_1^s \Gamma} \end{cases} \tag{S23}$$

On the other hand, the dislocation density as the function of plastic strain was derived from Equation S15 and is presented as Equation S24.

$$\sqrt{\rho} = \frac{k_1}{k_2} - \left(\frac{k_1}{k_2} - \sqrt{\rho_0}\right) \exp\left(-\frac{k_2}{2}\varepsilon\right) \tag{S24}$$

where ρ_0 is the initial dislocation density and it can be expressed from initial flow stress (Equation S25):

$$\tau_0^s = \alpha G b \sqrt{\rho_0} \tag{S25}$$

By substituting k_1, k_2 and ρ_0, which are expressed in terms of the Voce parameters, into Equation S25, the time-dependent formula of dislocation density during the creep deformation has been obtained, as shown in Eq. S26 as well as Equation S3 in Supplementary Note 1.

$$\sqrt{\rho} = \frac{\tau_0^s}{\alpha G b} + \frac{\tau_1^s + \theta_1^s \Gamma}{\alpha G b}\left[1 - \exp\left(-\frac{\theta_0^s}{\tau_1^s}\Gamma\right)\right] \tag{S26}$$

While XRD is widely employed for estimating dislocation densities, it is well established that this technique tends to overestimate dislocation content in materials with inherently low dislocation densities — such as our Mo-14Re alloy, which underwent sintering, deformation, and subsequent annealing. As shown in Figure 3b in the main text, the baseline dislocation density of the annealed alloy falls within a range where XRD-based analysis becomes unreliable due to overlapping contributions from grain size inhomogeneity and other microstructural features. As a practical interim approach, we qualitatively assessed dislocation evolution using TEM micrographs (Figures 3c-d in the main text). Although TEM observations are subject to underestimation — as extinction effects limit visibility to only a subset of dislocation segments — the micrographs unambiguously reveal a clear increase in dislocation density following creep deformation. This observed trend is fully consistent with the evolution predicted by Equation S3, providing qualitative experimental support for the modeled behavior.

References

[1] Turner, P. A., and Tomé, C. N., "Self-consistent modeling of visco-elastic polycrystals: Application to irradiation creep and growth," *Journal of the Mechanics and Physics of Solids* (1993): 1191, https://doi.org/10.1016/0022-5096(93)90090-3.

[2] Lebensohn, R. A., Turner, P. A., Signorelli, J. W., Canova, G. R., and Tomé, C. N., "Calculation of intergranular stresses based on a large-strain viscoplastic self-consistent polycrystal model," *Modelling and Simulation in Materials Science and Engineering* (1998): 447, https://doi.org/10.1088/0965-0393/6/4/011.

[3] Tome, C., Canova, G. R., Kocks, U. F., Christodoulou, N., and Jonas, J. J., "The relation between macroscopic and microscopic strain hardening in F.C.C. polycrystals," *Acta Metallurgica* (1984): 1637, https://doi.org/10.1016/0001-6160(84)90222-0.

[4] Nes, E., "Modelling of work hardening and stress saturation in FCC metals," *Progress in Materials Science* (1997): 129, https://doi.org/10.1016/S0079-6425(97)00032-7.

[5] Chen, X., Suzuki, T., Tom, P. P., et al., "Dispersed barrier hardening modeling on depth-distributed helium bubbles in iron-based alloys," *Journal of Nuclear Materials* (2025): 155608, https://doi.org/10.1016/j.jnucmat.2025.155608.

[6] Onaka, S., Huang, J. H., Wakashima, K., and Mori, T., "Stress relaxation caused by the combination of interfacial sliding and diffusion around spherical inclusions," *Mechanics of Materials* (1999): 717, https://doi.org/10.1016/s0167-6636(99)00033-2.

[7] Mori, T., Onaka, S., and Wakashima, K., "Role of grain-boundary sliding in diffusional creep of polycrystals," *Journal of Applied Physics* (1998): 7547, https://doi.org/10.1063/1.367519.

[8] Gu, Q., Wang, Y., Leng, E., Huang, Y., and Jin, N., "First-principles study of the stability and mechanical properties for binary Mo–Re alloys," *Physica B-Condensed Matter* (2024): 416401, https://doi.org/10.1016/j.physb.2024.416401.

[9] Was, G. S., *Irradiation Creep and Growth*, (Berlin: Springer, 2017), ISBN 9781493934386.

[10] Wang, F., Zhai, Z., Zhao, Z., Di, Y., and Chen, X., "Physics-informed neural network for lithium-ion battery degradation stable modeling and prognosis," *Nature Communications* (2024): 4332, https://doi.org/10.1038/s41467-024-48779-z.

[11] Wang, J., Han, C., Zong, H., Ding, X., and Sun, J., "Effect of Re segregation on irradiation damage behavior of Mo-Re alloys," *Computational Materials Science* (2025): 113704, https://doi.org/10.1016/j.commatsci.2025.113704.

[12] Smirnova, D., Starikov, S., Leines, G. D., et al., "Atomistic description of self-diffusion in molybdenum: A comparative theoretical study of non-Arrhenius behavior," *Physical Review Materials* (2020): 13605, https://doi.org/10.1103/physrevmaterials.4.013605.

[13] Plimpton, S., "Fast Parallel Algorithms for Short-Range Molecular Dynamics," *Journal of Computational Physics* (1995): 1, https://doi.org//10.1006/jcph.1995.1039.

[14] Kombaiah, B., Dasari, S., Bhave, C., et al., "Creep-Induced Elemental Redistribution at Grain Boundaries of 304L Stainless Steel – An Experimental Evidence for Diffusional Creep Mechanisms,"

Acta Materialia (2025): 121137, https://doi.org/10.1016/j.actamat.2025.121137.

[15] Andersson, J., and ågren, J., "Models for numerical treatment of multicomponent diffusion in simple phases," *Journal of Applied Physics* (1992): 1350, https://doi.org/10.1063/1.351745.

[16] Tripathi, P., and Milner, S. T., "Efficient simulations of mobility matrices for electrolytes by applying forces," *Chemical Science* (2024): 16176, https://doi.org/10.1039/d4sc03325f.

[17] Mishin, Y., and Cahn, J. W., "Thermodynamics of Cottrell atmospheres tested by atomistic simulations," *Acta Materialia* (2016): 197, https://doi.org/10.1016/j.actamat.2016.07.013.

[18] Thirathipviwat, P., Kotake, T., Suzuki, T., et al., "Effect of fine dispersoids on dislocation density and dislocation rearrangement of Al-Mn alloy during tensile deformation," *Materials Science and Engineering: A* (2025): 147997, https://doi.org/10.1016/j.msea.2025.147997.

[19] Xu, W., Maksymenko, A., Hasan, S., Meléndez, J. J., and Olevsky, E., "Effect of external electric field on diffusivity and flash sintering of 8YSZ: A molecular dynamics study," *Acta Materialia* (2021): 116596, https://doi.org/https://doi.org/10.1016/j.actamat.2020.116596.

[20] Tome, C. N., and Lebensohn, R. A., *Material Modeling with the Visco-Plastic Self-Consistent (VPSC) Approach*, (Amsterdam: Elsevier, 2023), ISBN 9780128207130.

[21] Mughrabi, H., "The α-factor in the Taylor flow-stress law in monotonic, cyclic and quasi-stationary deformations: Dependence on slip mode, dislocation arrangement and density," *Curr. Opin. Solid State Mater. Sci.* (2016): 411, https://doi.org/10.1016/j.cossms.2016.07.001.
